# Evaluation of the Antibacterial, Antioxidant, Anticancer, and Antidiabetic Activities of the Leaves and Inflorescences of *Crassula capitella*

**DOI:** 10.3390/biomedicines14010121

**Published:** 2026-01-07

**Authors:** Sahar Abdulaziz AlSedairy, Manal Abdulaziz Binobead, Fuad Alanazi, Ibrahim M. Aziz

**Affiliations:** 1 Department of Food Sciences and Nutrition, College of Food and Agricultural Sciences, King Saud University, P.O. Box 2460, Riyadh 11451, Saudi Arabia; mbinobead@ksu.edu.sa; 2 Department of Clinical Laboratory Sciences, College of Applied Medical Sciences, King Saud University, Riyadh 12372, Saudi Arabia; foalanazi@ksu.edu.sa; 3 Department of Botany and Microbiology, College of Science, King Saud University, P.O. Box 2455, Riyadh 11451, Saudi Arabia

**Keywords:** natural products, *Crassula capitella*, cancer, oxidative stress, therapy

## Abstract

**Background/Objectives**: Plants of the Crassulaceae family have been utilized in traditional medicine because of their medicinal properties. *Crassula capitella*, an ornamental succulent plant, has not yet received significant attention from physiochemists or pharmacologists. The objective of this study was to investigate the in vitro phytochemical properties and biological activity of methanolic extracts obtained from the leaves (CCLE) and inflorescences (CCIE) of *C. capitella*. **Methods**: Phytochemical screening included GC/MS analysis. The in vitro investigation of biological properties includes the assessment of antibacterial activity, utilizing disk diffusion assays and measuring MIC and MBC values for Gram-positive and Gram-negative bacteria. Antioxidant properties were determined through IC50 values in DPPH and ABTS assays. Cytotoxicity properties were evaluated using the MTT assay in MCF-7 and HepG2 cells, along with an analysis of apoptosis gene expression. Additionally, the antidiabetic effects were examined through α-amylase or α-glucosidase inhibition assays. **Results**: GC/MS analysis revealed distinct differences. CCLE contained more terpenoids such as betulinaldehyde (30.53%) followed by lupeol (19%) and betulin (4.07%), whereas CCIE was rich in fatty acids. The TPC and TFC of CCIE (88.17 mg GAE/g and 57 mg QE/g) were significantly greater than those of CCLE. Compared with CCLE, CCIE exhibited greater antibacterial properties (MIC values of 6.25 µg/mL toward *S. aureus*), greater antioxidant properties (IC_50_ values in the DPPH/ABTS assay), antitumor properties (IC_50_ values of approximately 90–96 µg/mL), and antidiabetic properties (IC_50_ values of 87–83 µg/mL in the α-amylase/α-glucosidase assay). Both bioactive extracts induced apoptosis in cancer cells by downregulating the expression of the tumorigenesis genes *bcl-2* and *bcl-xL*. **Conclusions**: The findings provided the first evidence about the evaluated the potential antibacterial, antioxidant, anticancer, and antidiabetic activities of *C. capitella*, which is attributed to its robust chemical composition and position it as a compelling candidate for further in vivo and sub-clinical applications.

## 1. Introduction

The genus *Crassula* is a member of the Crassulaceae family, which is very rich in succulents [1,2]. In the southern hemisphere, more than 200 species are scattered, which is especially prominent in South Africa [3]. Many *Crassula* species, particularly the commonly cultivated *Crassula ovata* (jade plant), have surpassed their ornamental value to be widely used in traditional medicine systems around the world [4]. *Crassula capitella* Thunb., known as Red Pagoda or Campfire, is a striking perennial with spiraled, lance-shaped leaves that blaze into vivid red under stress. *C. capitella* is popular as an ornamental, and medicinal uses have sometimes been suggested for related species; however, *C. capitella* itself has not been the subject of deep, rigorous scientific studies. Early phytochemical research revealed that it contains valuable compounds such as bergenin, kaempferol, and quercetin derivatives; however, much remains unknown [5]. Compared with other members of the Crassulaceae family, such as *Kalanchoe* and *Sedum*, which for centuries have been utilized in traditional medicine for the treatment of wounds, inflammation, and infections, the ethnopharmacological potential of *Crassula* remains almost unexploited, although its ornamental value is well appreciated worldwide [6,7].

Preliminary research on *Crassula* highlights a pool of bioactive metabolites waiting to be tapped. Investigations of closely related species, such as *C. ovata*, have revealed the presence of flavonoids, triterpenoids, and phenolic compounds, which are usually implicated in their antioxidant, antimicrobial, and anti-inflammatory activities [8]. However, we still lack a full phytochemical and pharmacological portrait of *C. capitella*. However, no previous study has comparatively assessed the bioactivities from different plant parts, leaves, and flowers against an array of targets, such as pathogenic bacteria, reactive free radicals, cancer cell lines, and enzymes linked with diabetes. These findings present a real opportunity for the scientific validation of traditional uses and new therapeutic agents.

We acknowledge the limitations and shortcomings of the methodology employed in this study. To validate the recently found chemicals, more research is required. It is necessary to utilize linear interpolation concerning the retention lengths of two common n-alkane mixes C_8_–C_20_ and C_21_–C_40_) in order to calculate the retention indices of the components. The study is therefore driven by the need to systematically unlock the medicinal potential of *C. capitella*, presenting a side-by-side look at its vegetative and reproductive organs. The main goals were to identify the major phytochemicals in the leaves and inflorescences of *C. capitella*, to quantify total phenolics and flavonoids, and to test their in vitro, antibacterial, antioxidant, anticancer, and antidiabetic effects. This work draws on clearly establishing the link between chemistry and bioactivity to provide scientific justification for the potential use of *C. capitella* in functional foods and the development of phytopharmaceuticals.

## 2. Materials and Methods

### 2.1. Preparation of the Leaves and Inflorescences of C. capitella

The leaves and inflorescences of *C. capitella* were collected from Ibb city in Yemen. It was found at King Saud University’s College of Science’s Herbarium in the Botany and Microbiology Department (KSU NO-345234). The leaves and inflorescences of *C. capitella* were thoroughly washed with distilled water and left to air-dry at room temperature before being ground into a powder via an electric mixer. Next, 10 g of plant powder was extracted with 100 mL of absolute methanol. Methanol is a highly soluble polar solvent that works effectively for extracting different polar substances. It is used to extract anthocyanins, terpenoids, lignans, polysaccharides, proteins, and amino acids in addition to phenolic chemicals, lipids, and fatty acids [9,10]. To remove contaminants and solid residues from the extract, Whatman No. 1 filter sheets were utilized. The extracts were then dried and concentrated via rotary vacuum evaporation (Yamato BO410, Yamato Scientific Co., Ltd., Tokyo, Japan). Finally, the dried extract was refrigerated at 4 °C for further use. The extract yield % was calculated via the following formula: yield (%) = weight of solvent-free extract (g) × 100/dried extract weight [11].

### 2.2. Determination of Bioactive Components

The bioactive components of the *C. capitella* leaf extract (CCLE) and the *C. capitella* inflorescence extract (CCIE) were identified using gas chromatography/mass spectrometry GC/MS (Santa Clara, CA, USA) via an Agilent Technologies system. In brief, 1.5 µL of the filtrate was injected via an autosampler injection system with an Agilent Technologies GC/MS 7890B GC system (Santa Clara, CA, USA) after 2 mg of each dried extract was dissolved in 2 mL of high-performance liquid chromatography (HPLC)—grade methanol and filtered through a 0.22 µm PTFE membrane filter. The products were identified via National Institute of Standards and Technology (NIST) database-integrated software (version 2.2). The identification of the sample components was achieved via gas chromatography coupled with a mass selective detector (GC/MS). For the separation of target compounds, a DB-5 MS fused silica capillary column (30 m × 0.25 mm, 0.25 μm) was used, with helium as the carrier gas at 1 mL/min (for 1 min). The oven temperature program began with 3 min at 40 °C, which was increased by 7.5 °C per minute to 280 °C (held for 5 min) and then to 290 °C (held for 1 min). The injector and detector temperatures were set to 200 °C and 300 °C, respectively. Data were collected in electron impact (EI) mode at 70 eV, scanning *m*/*z* 91–283. The split injection ratio was 1:10 (1 μL volume), and the total run time was 60 min. The MS detector was set as follows: acquisition scan type, mass ranging from 40 to 500 g/mol, scan speed of 1.56, 8 min solvent delay, and 230 °C MS source temperature. The compounds were identified by comparing their spectra with those of the Wiley and NIST mass libraries, considering matches above 90%. determined. This qualitative profiling was quantified using relative peak area percentages. The relative abundance of each identified compound is expressed as a percentage of the total integrated chromatogram area, as determined by the instrument’s data system (ChemStation) without the use of an internal standard.

### 2.3. Determination of Total Phenolic Content (TPC) and Total Flavonoid Content (TFC)

The TPC values of CCLE and CCIE were estimated via the Folin–Ciocalteu method with slight modifications [12]. In brief, 0.1 mL of the extract (1 mg/mL) was mixed with 1 mL of diluted Folin–Ciocalteu reagent, and 1.5 mL of distilled water was added. The mixture was left for 5 min, after which 1 mL of sodium carbonate (Na_2_CO_3_) (7.5 g/100 mL solution) was added. After incubation at 25 °C for 30 min, the absorbance of the resulting blue color was measured at 765 nm via a spectrophotometer (U2001 U2001 UV–VIS-Spectrophotometer, Hitachi, Tokyo, Japan). Gallic acid was used as a standard compound. The TPC was then calculated using the equation (y = 0.006x + 0.145, where co-efficient (R^2^) = 0.993 for the CCLE and y = 0.005x + 0.0235, and R^2^ = 0.992 for CCIE, derived from established standard curve with the gallic acid equivalent (GAE) and the results are presented as milligrams of gallic acid equivalents per gram of dry weight (mg GAE/g DW).

The TFC values of CCLE and CCIE were determined as described by [13], with minor modifications. Briefly, 1 mL of 2% aluminum chloride (AlCl_3_) was mixed with 500 μL of CCLE or CCIE (1 mg/mL). The mixture was then mixed with 3 mL of sodium acetate solution (50 mg/L). The absorbance at 420 nm was subsequently measured following 1 h of incubation at room temperature, and a blank sample was used as a reference (comprising 2 mL of sample solution and 2 mL of methanol devoid of AlCl_3_) via a spectrophotometer (U2001 UV–VIS-Spectrophotometer, Hitachi, Japan). A standard curve derived from the quercetin equivalent (QAE) standard was used to compute the TPC. The standard curve equation for the CCLE was (y = 0.0016x + 0.141 with R^2^ = 0.992), and for CCIE (y = 0.0023x + 0.0391, R^2^ = 0.989), and the TFC was expressed as quercetin equivalents in milligrams per gram of dry sample (mg QE/g DW).

### 2.4. Antibacterial Activity

#### 2.4.1. Disc Diffusion Method

The antibacterial activity of CCLE and CCIE was assessed via a disc diffusion assay as previously described [14] against 3 Gram-positive bacteria: *Staphylococcus aureus* (MTCC-29213), *Staphylococcus epidermidis* (MTCC-12228), *Bacillus subtilis* (MTCC-10400) and 3 Gram-negative bacteria: *Escherichia coli* (ATCC-25922), *P. aeruginosa* (MTCC-27853), and *Klebsiella* (MTCC-13883). Muller–Hinton broth (MHB) was employed for the cultivation of the bacteria being studied, which were incubated for 24 h at 37 °C. Subsequently, Mueller–Hinton agar (MHA) was combined with 0.1 mL of bacterial mixture, maintaining a McFarland turbidity of 0.5. A sterile cork borer was used to create 5 mm-diameter holes at consistent intervals to form wells. Various concentrations of CCLE and CCIE (100, 200, 400, and 800 µg/mL) were subsequently added to the wells. Chloramphenicol (25 µg/ mL) served as the positive control, while Muller–Hinton broth (HMB) was used as the negative control. All the plates were incubated at 4 °C for two hours, followed by incubation at 37 °C for twenty-four hours to promote microbial growth and assess the zone of inhibition surrounding each well. The millimeters (mm) of the inhibitory zone that formed around the discs were measured.

#### 2.4.2. Determination of Minimum Inhibitory Concentration (MIC) and Minimum Bactericidal Concentration (MBC) Values

The MICs of CCLE and CCIE were assessed against the bacterial strains previously mentioned via the microdilution broth method in a 96-well microplate. The assay incorporated 2,3,5-triphenyltetrazolium chloride (TTC). Two hundred microliters of MHB medium were added to every well of the microplate. CCLE and CCIE were examined via twofold serial dilutions from 1.56 to 800 µg/mL. Chloramphenicol at 25 µg/mL served as the positive control for the MIC assay. After the bacterial cell mixture was adjusted to 10^6^ CFU/mL, 10 µL was added to each well. The microplates were incubated at 37 °C for 24 h. Then, 20 µL of TTC (2 mg/mL) was added to each well. The appearance of a red color indicated bacterial proliferation. The lowest concentration with no observable color change was recorded as the MIC. To determine the MBC, 100 µL from wells without color changes were cultured on MHA and incubated at 37 °C for another 24 h [15].

### 2.5. Antioxidant Activity

#### 2.5.1. 2,2-DDiphenyl-1–Picrylhydrazyl (DPPH) Radical Scavenging Activity

The DPPH scavenging activity of CCLE and CCIE was determined as described by [16]. Various concentrations of CCLE and CCIE were prepared (100, 200, 400, and 800 µg/mL). To each concentration of the extract, 2 mL of freshly prepared DPPH solution (0.06% *w*/*v*) prepared in methanol was added, and the mixture was vigorously agitated. A commercial antioxidant (ascorbic acid) (100–800 μg/mL) was used as the standard control. The reaction mixtures were vortexed and left to stand at room temperature in the dark for 30 min. After the incubation period, the optical density (OD) of the samples was measured at 517 nm via a U2001 UV–VIS (U2001 (Hitachi, Japan)). The IC_50_ values for ascorbic acid and CCLE and CCIE, indicating the concentration required to reduce the initial DPPH concentration by 50%, were determined via Graph Pad Prism software (version 5.0, La Jolla, CA, USA). The ability to scavenge the DPPH radical was calculated via the following formula: DPPH radical scavenging activity (%) = (OD of control − absorbance of the extract)/OD of control × 100.

#### 2.5.2. 2,2′-Azino-Bis (3-Ethylbenzothiazoline-6-Sulfonic Acid (ABTS)) Assay

The ABTS^+^ test of the CCLE and CCIE was carried out as described earlier [17]. The concentrations of ascorbic acid, CCLE, and CCIE were 100, 200, 400, and 800 µg/mL, respectively. The ABTS solution, consisting of 192 mg of solution dissolved in 50 mL of distilled water, was combined with the 140 mM solution. The ABTS solution (192 mg in 50 mL of distilled water) was mixed with 140 mM K_2_S_2_O_8_ solution (K_2_S_2_O_8_) in the dark for 12–16 h at 25 °C to generate the ABTS cation radical (ABTS^+^). This radical mixture was subsequently diluted in methanol (1:89, *v*/*v*) to obtain an OD of approximately 0.70 ± 0.02 at 734 nm. The assay involved mixing 1 mL of the diluted ABTS^+^ solution with 1 mL of each concentration of CCLE and CCIE or ascorbic acid. A spectrophotometer was used to measure the OD at 734 nm after the reaction mixtures were allowed to equilibrate at 30 °C. The readouts of the ABTS^+^ % and IC_50_ values are presented as described above.

### 2.6. Cell Culture and Cytotoxicity Assays

The cytotoxic effects of methanolic extracts obtained from CCLE and CCIE were evaluated in human hepatoma HepG2 (ATCC HB-8065) and breast cancer MCF-7 (ATCC HTB-22) cell models [18,19]. The cells were maintained in Dulbecco’s modified Eagle’s medium (DMEM) supplemented with sodium pyruvate, L-glutamine, glucose (4.5 g/L), and fetal bovine serum at a final concentration of 10.0%. The 3-(4,5-dimethylthiazol-2-yl)-2,5-diphenyl-2H-tetrazolium bromide (MTT) assay was utilized to determine cell viability [20]. To prevent contamination of the medium, it was further supplemented with 1.0% antibiotics (100 U/mL penicillin and 1000 μg/mL streptomycin). Various concentrations of CCLE and CCIE extracts (100, 200, 400, and 800 µg/mL) were added to the culture medium, and the cells were incubated for 24 h at 37 °C with 5% CO_2_. The positive control was cisplatin (30 µg/mL). The cells were not exposed to CCLE or CCIE, which served as negative controls. After incubation, 10 µL of the MTT solution (5 mg/mL) was added to each well. The plates were incubated for 2 to 4 h. An equal volume of 1:1 (200 μL) dimethyl sulfoxide (DMSO) and isopropanol mixture was added to each well and incubated for 30–45 min. Cell proliferation was detected by measuring the absorbance of each well at 590 nm using an ELX-808 microplate reader (BioTek Laboratories, LLC, Shoreline, WA, USA), with a reference wavelength of 620 nm. The percentages of cell viability and cell death were calculated using the following formulas:

The cell viability (%) = [(OD of treated cells − absorbance of the extract)/OD of untreated cells (control)] × 100. GraphPad Prism software (version 5.0, La Jolla, CA, USA) was used to calculate the IC_50_ values, with the mean values ± SD used for data processing [21].

### 2.7. Antidiabetic Activity

#### 2.7.1. Determination of α-Amylase Inhibitory Activity

In vitro α-amylase inhibition by CCLE and CCIE was studied via the 3,5-dinitrosalicylic acid (DNSA) method [22]. In summary, the plant extracts were diluted to achieve concentrations between 50 and 1000 μg/mL with a buffer solution (0.02 M Na2HPO4/NaH2PO4; 0.006 M NaCl; pH 6.9). The mixture was incubated for 10 min at 37 °C after combining 200 µL of each extract with 200 µL of the Molychem α-amylase solution (2 units/mL). Each tube was subsequently filled with 200 µL of a 1% starch solution (*w*/*v*) and incubated at 37 °C for 3 min. To halt the reaction, 200 µL of DNSA reagent (composed of 12 g of sodium potassium tartrate tetrahydrate in 8.0 mL of 2 M NaOH and 20 mL of 96 mM 3,5-DNSA solution) was added, followed by heating for 10 min at 85 °C in a water bath. The positive control consisted of 100 μL of 400 µg/mL acarbose (Bayer, Berlin, Germany). After the sample was allowed to cool to room temperature and diluted with 5 mL of distilled water, the optical density at 540 nm was recorded using a U2001 UV–VIS spectrophotometer (U2001 UV–VIS spectrophotometer, Hitachi, Japan). The following formula was used to calculate the percentage inhibition of α-amylase.

The inhibitory activity of the extract (%) = [(X − Y)/X] × 100, where X denotes the reaction occurring without the extract, whereas Y indicates the increase in absorbance when the extract is present. The IC_50_ values were determined via GraphPad Prism software (version 5.0; La Jolla, CA, USA).

#### 2.7.2. Determination of α-Glucosidase Inhibitory Activity

The α-glucosidase inhibitory activity of CCLE and CCIE was measured via yeast α-glucosidase and p-nitrophenyl-α-D-glucopyranoside (pNPG) as previously reported [23]. To obtain 0.5 to 5.0 mg/ mL final concentrations, 50 μL of α-glucosidase (1 U/ mL) produced in 0.1 M phosphate buffer (pH 6.9) and 250 μL of 0.1 M phosphate buffer were added to the CCLE and CCIE or acarbose (a positive control) (100 μL of 2 to 20 mg/ mL). The mixture was preincubated for twenty minutes at 37 °C. Ten microliters of 10 mM pNPG in 0.1 M phosphate buffer (pH 6.9) was added after preincubation and incubated for 30 min at 37 °C. The reactions were terminated by the addition of 650 μL of 1 M sodium carbonate, and the absorbance was measured at 405 nm via a UV–vis spectrophotometer (U2001 UV–vis Spectrophotometer, Hitachi, Japan). The percentage of inhibition of enzyme activity and the IC_50_ values were calculated as described above.

#### 2.7.3. Statistical Analysis

All values are expressed as the means ± SDs. Statistical difference and linear regression analyses were performed via GraphPad Prism software (version 5.0; La Jolla, CA, USA), with *p* ≤ 0.05 considered statistically significant. All measurements were performed in triplicate.

## 3. Results

### 3.1. Extraction Yields

The yields of the CCLE and CCIE were 12.48% and 14.12%, respectively, on the basis of the applied operating mode and the dry matter weight computation (*w*/*w*).

### 3.2. Chemical Composition of the CCLE and CCIE

**Figure 1:**
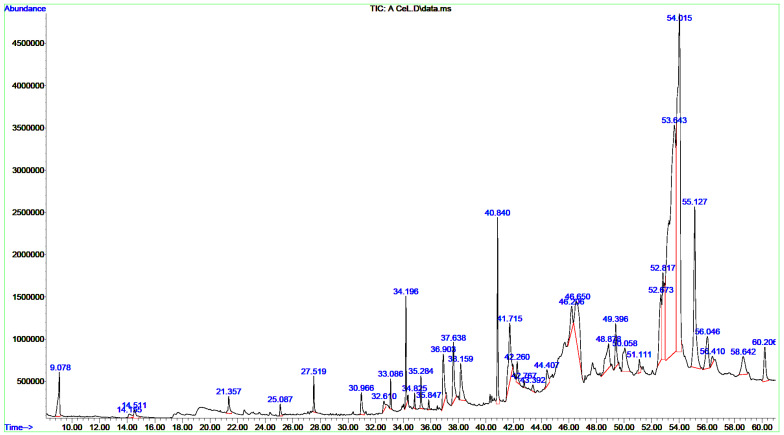
GC/MS chromatograms of CCLE. All spectral peaks correlate with the identified chemicals, with a major peak indicating the primary constituent of the extract.

**Figure 2:**
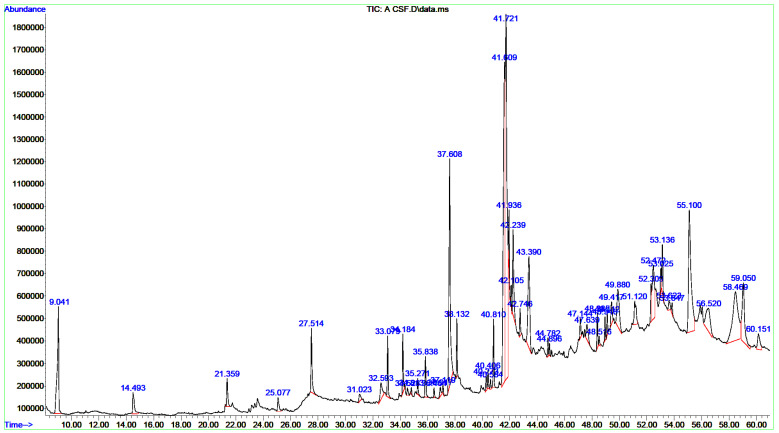
GC/MS chromatograms of CCIE. A prominent peak in the spectrum indicates the major component of the extract, and each peak corresponds to a recognized chemical.

A single GC-MS analysis of both CCLE and CCIE revealed remarkably identical chromatographic profiles with Willey and NIST mass libraries, accounting for matches greater than 90%. The GC–MS chromatograms of these bioactive compounds are displayed in Figure 1, with their peak retention time (RT), peak areas (%), molecular formula (MF), and molecular weight (MW) listed in Table 1. Thirty-seven peaks in total were recorded for the bioactive components of CCLE, which were identified by comparing their RT, peak area (%), MF, and MW to those of the known compounds listed in the NIST library. The CCLE exhibited a pronounced accumulation of several pentacyclic triterpenoids: betulinaldehyde (30.53%) followed by lupeol (19%) and betulin (4.07%) (Table 1 and Figure 1). However, a total of 51 bioactive components were discovered in the CCIE, with cis-9-tetradecenoic acid isobutyl ester (16.27%) being the predominant compound, followed by cis-linoleic acid (14.45%), and palmitic acid (n-hexadecanoic acid) (7.83%) (Table 2 and Figure 2).

### 3.3. TPC and TFC of the CCLE and CCIE

The TPC and TFC assay results revealed a significant difference in phytochemical content between CCLE and CCIE. The TPC of CCIE (88.17 mg GAE/g DW) was higher than that of the CCLE (64.15 mg GAE/g DW). Similarly, the TFC for the CCIE was found to be 57 mg QE/g DW higher than that of the CCLE (49 mg QE/g DW).

### 3.4. Antibacterial Effects of CCLE and CCIE

The disc diffusion technique was used to assess the antibacterial properties of the methanol extracts of CCLE and CCIE against various bacterial strains. Table 3 and Table 4 present the ability of this extract to prevent the growth of the tested bacteria. The findings demonstrated that the extracts inhibited the growth of bacterial strains in a dose-dependent manner at various concentrations. The antibacterial activity of CCLE and CCIE increased gradually with increasing concentration; at 400 and 800 μg/mL, the inhibitory zones began to increase significantly (*p* < 0.05), although not as much as those of the positive control (25 µg/mL chloramphenicol). Compared with CCLE, CCIE had greater antibacterial activity, with an MIC of 6.25 ± 0.00–25 ± 0.00 μg/mL, than CCLE, with an MIC of 6.25 ± 0.00–50 ± 0.00 μg/mL. Gram-positive bacteria, particularly *S. aureus*, were more vulnerable to CCLE and CCIE.

### 3.5. DPPH and ABTS+ Radical Scavenging Activity

**Figure 3:**
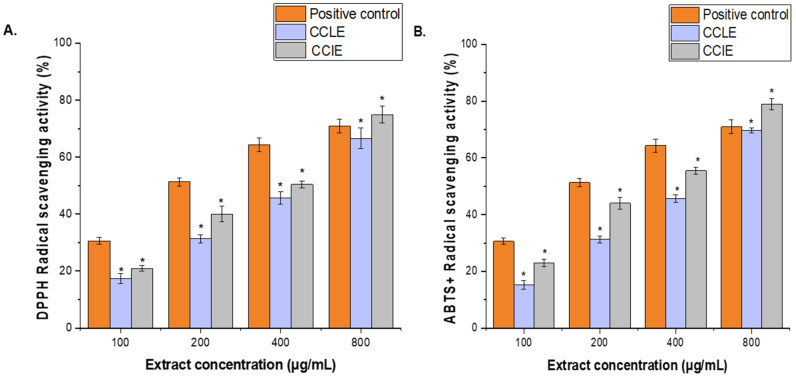
Antioxidant activity of CCLE and CCIE. (**A**), DPPH reducing power and (**B**), ABTS^+^ scavenging activity at various concentrations (100–800 μg/mL). Ascorbic acid (100–800 µg/mL) was used as a positive control. The mean value of 3 independent experiments is presented. The scavenging activity of CCLE and CCIE was significantly lower (*) than that of the positive control at a significance level of *p* < 0.05. + = radical cation.

Two distinct techniques were used to examine the phytochemicals CCLE and CCIE’s antioxidant activity. Results of the DPPH and ABTS+ tests’ radical scavenging activity were shown in Figure 3A,B. Ascorbic acid (100–800 μg/mL) was used as a standard antioxidant to compare the results. Both the ABTS and DPPH techniques showed better antioxidant activity at higher extract concentrations. At every dosage point except 800 μg/mL, ascorbic acid demonstrated a greater level of free radical-scavenging activity than the plant extract. The percentage of CCLE and CCIE scavenging activity was 64.66 ± 0.79% and 75.43 ± 1.52%, respectively, at the highest concentration of 800 μg/mL, whereas the percentage of ascorbic acid scavenging activity was 75.69 ± 2.25% at the same concentration. Additionally, CCLE and CCIE had IC_50_ values of 95.41 ± 0.18 μg/mL and 112.32 ± 1.17 μg/mL, respectively, whereas ascorbic acid had an IC_50_ value of 29.15 ± 0.11 μg/mL (Figure 3A).

Similarly, at the maximum concentration of 800 μg/mL, the percentage of CCLE and CCIE scavenging activity was 69.83 ± 0.9% and 78.92 ± 1.94%, respectively, while the percentage of ascorbic acid scavenging activity was 70.99 ± 2.45%. Whereas, the IC_50_ values for ascorbic acid, CCIE, and CCLE were 26.12 ± 2.18 μg/mL, 82.71 c± 1.31 μg/mL, and 126.31 ± 2.18 μg/mL, respectively, (Figure 3B).

### 3.6. Cell Cytotoxicity

**Figure 4:**
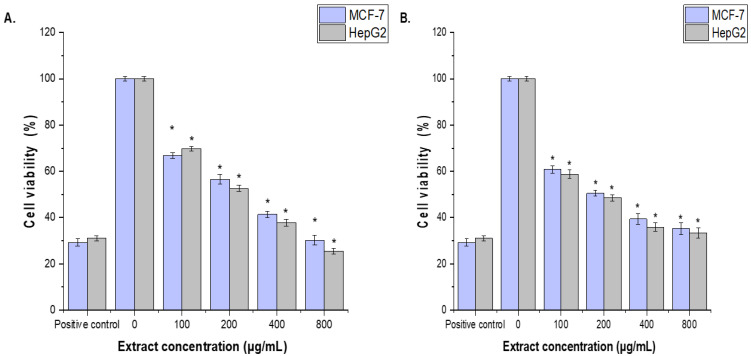
Effects of (**A**), CCLE and (**B**), CCIE on MCF-7 and HepG2 cell viability, as determined via the MTT assay. The cells were treated with CCLE or CCIE (100, 200, 400, or 800 µg/mL) for 48 h. The mean values ± SD of three independent experiments are shown. (* = *p* < 0.05 compared with nontreated cells (negative control)).

The cytotoxic effects of CCLE and CCIE on MCF-7 and HepG2 cells were assessed via the MTT assay. The anticarcinogenic properties of CCLE and CCIE were tested at concentrations of 100, 200, 400, and 800 µg/mL. The cytotoxic effects of CCLE and CCIE on MCF-7 and HepG2 cells were concentration dependent. Compared with that of the untreated control cells, the proliferation of the MCF-7 and HepG2 cell lines treated with 100 μg/mL CCLE and CCF was significantly (*p* < 0.05) inhibited, although it was less than that of the positive control (30 µg/mL cisplatin). The CCIE showed higher cytotoxic impacts with (IC_50_ = 96.14 ± 1.18 μg/mL and 90.12 ± 0.13 μg/mL against MCF-7 and HepG2 cells, respectively, than those of CCLE, the IC_50_ = 136.12 ± 2.12 μg/mL and 118.95 ± 3.21 μg/mL, respectively (Figure 4A,B).

**Figure 5:**
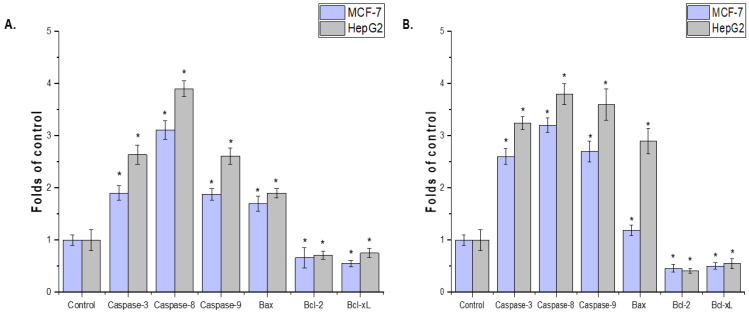
The effects of (**A**), CCLE and (**B**), CCIE on MCF-7 and HepG2 cells and determination of pro- and antiapoptotic marker genes (caspase-3, 8, and 9, Bax, Bcl-2, and *Bcl-Xl* genes). The values represent the means ± SDs from ± trials (* = *p* < 0.05).

**Figure 6:**
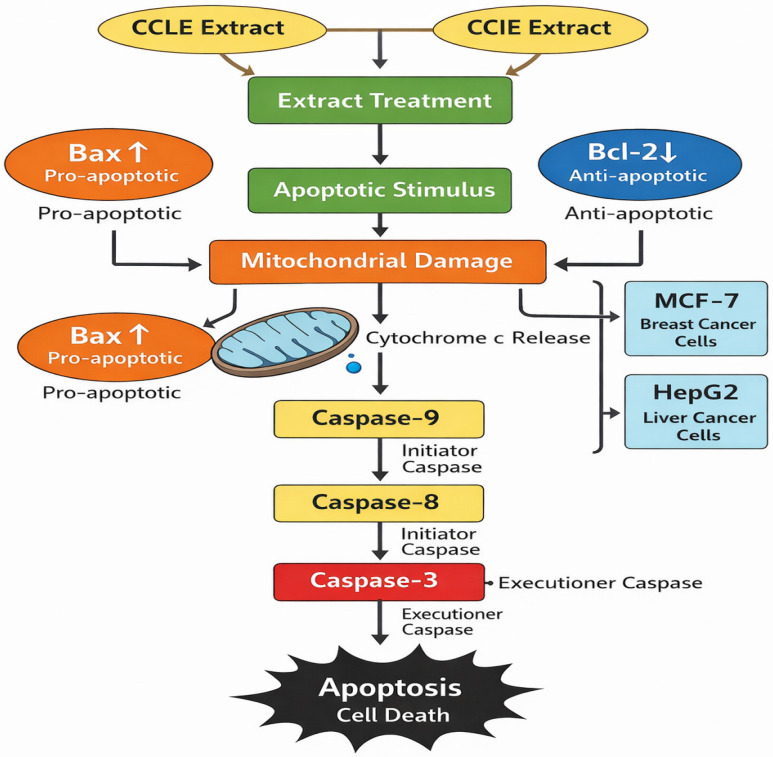
The diagram of mechanism of action of apoptosis after treatment with CCLE and CCIE on MCF-7 and HepG2 cells.

The effects of CCLE and CCIE on HepG2-induced apoptotic signaling and MCF-7 cells were evaluated. The MCF-7 and HepG2 cell lines treated with the seed extract presented increased levels of mRNA expression, according to the results of the rRT–PCR study. The expression of antiapoptotic genes (Bcl-xL and Bcl-2) was lower in the MCF-7 and HepG2 cell lines treated with the plant extract than in the control group (*p* < 0.05) (Figure 5A,B). The possible mechanism of apoptosis after CCLE and CCIE treatment of MCF-7 and HepG2 cells is depicted in Figure 6.

### 3.7. In Vitro Antidiabetic Activities of CCLE and CCIE

**Figure 7:**
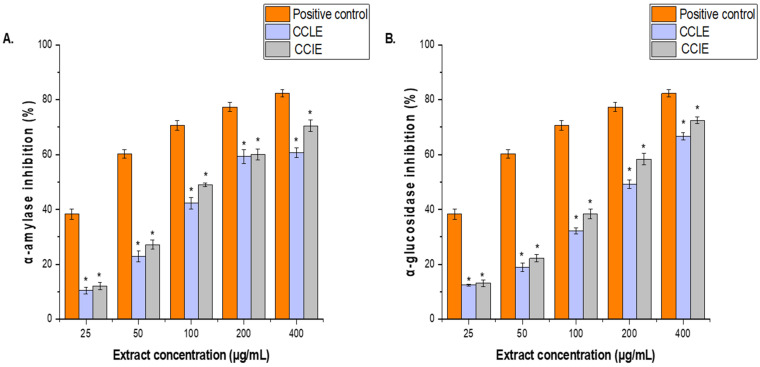
Effects of (**A**), CCLE and (**B**), CCIE on α-amylase and α-glucosidase inhibitory activities at various concentrations (25–400 μg/mL). The results are the mean values of three replicates. The results are presented as the means ± SDs of three experiments (* = *p* < 0.05 compared with the acarbose positive control).

The antidiabetic activities of CCLE and CCIE were determined on the basis of their inhibitory effects on *α*-amylase and *α*-glucosidase enzymatic activities. The results are expressed as IC_50_ values. CCLE strongly inhibited α-amylase and α-glucosidase (IC_50_ = 104 ± 1.84 μg/mL and 97.61 ± 1.16 μg/mL, respectively) (Figure 7A), whereas CCIE inhibited α-amylase (IC_50_ = 87 ± 2.14 μg/mL) and α-glucosidase (IC_50_ = 83.62 ± 1.42 μg/mL) (Figure 7B). Conversely, both CCLE and CCIE presented IC_50_ values for the two enzymes that were less than those of the positive control, which was noted.

## 4. Discussion

The extraction of bioactive compounds is the most important initial step in the study of medicinal plants and is necessary to determine their pharmacological potential. In this context, CCLE and CCIE presented moderate to good recovery yields of soluble phytochemicals from this species: CCLE (12.48%) and CCIE (14.12%). This extractive yield demonstrates the efficacy of the methanol-based maceration process in solubilizing a significant amount of the plant’s phytochemicals. This finding is consistent with the literature on the Crassulaceae family, which is known to contain many phenolic, flavonoid, sterol, and terpenoid classes that methanol tends to extract well [2,6]. For example, *C. ovata* has been reported to possess similar classes of phenols and flavonoids [4]. Later in this discussion, the subsequent phytochemical analysis will indicate the presence of different bioactive metabolites. Compounds like bergenin and its derivatives are known for their antioxidant, anti-inflammatory, and antidiabetic properties, which present a well-defined chemical justification for the observed biological activities under investigation. Although direct, detailed comparisons of extraction yield from Middle Eastern flora are not available for *C. capitella* in the literature, regional methodological research on medicinal plants highlights relevant factors. That includes the type of solvent and technique, whether maceration or decoction, which significantly affect the yield of and chemical composition of the recovered phytochemicals, supporting the chosen ethanol-based extraction approach [24,25,26].

GC/MS profiling of the ethanolic extracts of the *C. capitella* leaves and flowers revealed a complex phytochemical profile, mainly composed of terpenoids, fatty acids, and their derivatives, along with steroids, with chemical compositions matching well with the chemical fingerprints reported for Crassulaceae. The CCLE exhibited a pronounced accumulation of several pentacyclic triterpenoids: betulinaldehyde (30.53%), lupeol (19.78%), and betulin (4.07%). Other major components include oleamide (6.57%), γ-sitosterol (4.22%), and vitamin E (2.96%), which give the leaves a peculiar chemical fingerprint. On the other hand, CCIE is characterized mainly by fatty acids and their esters, dominated by cis-9-tetradecenoic acid isobutyl ester (16.27%), cis-linoleic acid (14.45%), and palmitic acid (n-hexadecanoic acid) (7.83%). The other significant compounds in flowers include oleamide (7.99%), stigmasterol (6.78%), and β-terpinyl acetate (4.07%). A sharp separation in the main classes of compounds in the two organs might demonstrate organ-specific biosynthesis and metabolite accumulation. This might indicate that leaves and flowers could have different therapeutic potentials.

The high representation of terpenoids, particularly derivatives of lupeol and betulin in CCLE, is in good agreement with the *Crassula* genus. A previous study revealed that the leaf extract of *Kalanchoe crenata* Haw. (*Crassulacea*) is rich in polysaccharides, terpenes, alkaloids, and flavonoids [27]. Another study involving GC/MS analysis of the methanolic extracts of three *Echeveria* species (*Crassulaceae*) revealed that they were rich in γ-sitosterol and lupenone [28]. Additionally, a study on *C. ovata* reported that terpenoids and flavonoids are among the major bioactive compounds [4,8]. Indeed, a focused phytochemical investigation of *C. capitella* itself reported the presence of fourteen phenolic compounds, including bergenin, kaempferol, and quercetin derivatives [5]. Our GC/MS method did not emphasize these phenolics, likely because the technique favors volatile and semivolatile substances, and thus, these substances are often more clearly detected by HPLC methods commonly used for phenolics. In any case, the co-occurrence of triterpenoids, phenolics, and sterols is typical for this genus. Of particular interest are the representative contents of lupeol, stigmasterol, and betulin, whose anti-inflammatory, anticancer, hepatoprotective, and antidiabetic properties have been well documented in pharmacological studies [29,30,31,32,33]. Therefore, the prominence of lupeol, betulin, and stigmasterol in the extracts of *C. capitella* is directly related to the traditional uses of these plants, with plausible modern therapeutic mechanisms.

In the current investigation, the CCIE presented slightly higher values for TPC and TFC than did the CCLE. This finding is compatible with the GC/MS results, which emphasized that leaves are richer in certain high-molecular-weight triterpenoids, such as lupeol and betulin. A pattern that seems to emerge is one of different metabolite pools, with flowers concentrating polar phenolic antioxidants and leaves accumulating more nonpolar terpenoids. Such an organ-specific division of secondary metabolites could mean that the overall bioactivity of the plant would vary considerably with the part used for extraction. Furthermore, compared with CCLE, CCIE had the lowest scavenging activity (IC_50_ = 95.41 μg/mL for DPPH and 82.71 μg/mL for ABTS^+^ radicals) (IC_50_ = 112.32 for DPPH and 126.31 μg/mL for DPPH and ABTS), reflecting stronger antioxidant action. This observation is consistent with previous results showing a much greater TPC/TFC in CCIE than in CCLE. Phenolics are well-known electron donors and thus constitute one of the major radical scavengers.

The TPC and TFC values and antioxidant activity position *C. capitella* in a competitive range with other medicinal plants of the Crassulaceae family and arid-region flora. A study conducted by Mbhele and colleagues (2022) compared six species of the Crassulaceae family and reported that the acetone extract of *C. capitella* had the highest TPC values and exhibited robust antioxidant activity compared with extracts of *Agapanthus inapertus, Cheilanthes hirta, Eriospermum flagelliforme, Euphorbia clavarioides*, and *Pelargonium alchemilloides* [34]. Other studies have shown that the methanolic extracts of some *Kalanchoe* species have antioxidant activities, while they are rich in phenolic compounds such as quercetin, kaempferol, bryophyllol, stigmasterol, and campesterol [35,36,37]. Additionally, a previous study revealed the strong antioxidant activity of *Callisia repens* (turtle vine) and *Crassula ovata* (jade), with IC50 values of 48.8% and 470.3%, respectively [38]. Hence, the remarkable radical-scavenging activity, in addition to the results of the GC/MS analysis, justifies the presence and action of these compounds. Additionally, other nonphenolic compounds, such as vitamin E (tocopherol) and various sterols in both extracts, may contribute to other antioxidant pathways, possibly in the prevention of lipid membranes against peroxidation [39]. These results support the notion that the plant may be useful in the management of oxidative stress–linked conditions such as diabetes, cancer, and inflammation.

Both extracts demonstrated robust, broad-spectrum antibacterial activity against a diverse range of Gram-positive and Gram-negative bacteria. Growth inhibition is dose dependent, with CCIE and CCLE having minor MICs of 6.25–25 μg/mL and 12.5–50 μg/mL, respectively. The most affected strains were notably the Gram-positive bacteria, where *S. aureus* showed the highest susceptibility, with an MIC of 6.25 μg/mL for CCIE. In general, CCIE exhibited stronger activity, probably due to its higher TPC, suggesting that phenolic and flavonoid compounds play a major role in the antibacterial activity observed. This substantial effect could be attributed to the high phytochemical content of bergenin, kaempferol, and quercetin derivatives, which have been shown to damage bacterial cell membranes and inhibit virulence factors. Furthermore, the presence of broad-spectrum activity, including against tough Gram-negative strains such as *E. coli* and *P. aeruginosa*, suggests that multitarget mechanisms may be aided by synergistic interactions among extract constituents, such as fatty acids and terpenoids identified via GC/MS analyses. The antibacterial profile of *C. capitella* is consistent with the known bioactivity of other Crassulaceae family members. In the study conducted by Lopez-Angulo et al. (2019), different species of *Echeveria* exhibited high antibacterial activity (MICs ≤ 1 mg/mL) against *S. aureus* and *E. coli* [40]. Other studies have shown similar antibacterial activities of *Orostachys cartilaginous* [41], *Orostachys japonicus* [42], and *Sedum aizoon* [43]. The stronger effect on Gram-positive bacteria probably comes from the lack of an outer membrane in those organisms, making them more susceptible to the disruptive action of phenolics and lipophilic compounds [44] in the extracts, such as the fatty acids abundant in CCIE and terpenoids in CCLE.

In the present study, the extracts of *C. capitella* highlighted their potential in addressing two major global health challenges, cancer and diabetes mellitus, through their cytotoxic and antidiabetic effects. MTT analysis revealed that CCLE and CCIE displayed clear, dose-dependent toxicity to the human breast adenocarcinoma (MCF-7) and hepatocellular carcinoma (HepG2) lines. The CCIE had IC_50_ values of approximately 90–96 μg/mL, whereas the CCLE had higher IC_50_ values. Notably, HepG2 cells showed greater sensitivity, suggesting a significant effect on liver cancer. For mechanistic insights at the molecular level, gene expression data further confirmed this cytotoxicity by demonstrating a large downregulation of two important antiapoptotic genes, Bcl-2 and Bcl-xL, in treated cells. This reduction in survival signals suggests that the primary mechanism of this cytotoxic action is mitochondria-mediated apoptosis. The pro-apoptotic profile of CCLE agrees with what is known for the major phytochemicals in CCLE, which are rich in triterpenoids such as lupeol and betulin, with well-documented apoptotic and anti-proliferative activities in numerous types of cancers [45,46]. The increased sensitivity of HepG2 cells could suggest the high metabolic function of the liver, which would make these cells particularly sensitive to the disruption of metabolic pathways through such compounds.

This observation that plant reproductive parts, such as flowers, have relatively high levels of bioactivity has also been observed in other species. Research has shown that certain Crassulaceae family members, including *Sedum aizoon* L., *Bryophyllum laetivirens*, and *Bryophyllum pinnata*, have anticancer effects on HepG2, MCF-7, A549, and cervical cancer cells [47,48,49].

The mechanistic finding that the downregulation of antiapoptotic genes (Bcl-2 and Bcl-xL) in the treated cells illustrates an intervention that promotes the mitochondrial pathway of apoptosis. This mechanism of intervention serves as an attractive aspect that most tested anticancer plant extracts would seek and, if achieved, would represent an effective anticancer strategy. Although the present IC_50_ values for *C. capitella* are high, well into the micrograms, they represent an important starting point. In context, research conducted on some of the most active medicinal plants available, such as fireweed (*Chamaenerion angustifolium*), indicates that the IC_50_ values, such as rutin concentrations, are as low as 0.28 mg/g for some cell lines [50]. This finding illustrates the important component of downstream processing that *C. capitella* needs to provide as an extract, as it provides the most active compounds for the promotion of apoptosis.

The strong inhibitory effects of CCLE and CCIE against α-amylase and α-glucosidase enzymes are major findings for the control of postprandial hyperglycemia and are the focus of concern in the management of type 2 diabetes. Once again, CCIE was more potent, with IC_50_ values of 87 µg/mL for α-amylase and 83.62 µg/mL for α-glucosidase. Although the extracts were less active than the commercial positive control drug acarbose was, the activity exhibited potential. Efforts to find new α-glucosidase inhibitors of plant origin are well underway, mainly for the purpose of finding new drugs that are less toxic than the available medicines. The fact that the extracts inhibited enzymes indicates that *C. capitella* has the potential to manage blood glucose levels. Similarly, different Crassulaceae plants exhibit antihyperglycemic activity. *Rhodiola rosea* (10 mg/paw) has the same effect as gabapentin (20 mg/paw) in reducing hyperalgesia and allodynia in diabetic rats [51]. *Sedum dendroideum* (SD) has been shown to have in vitro hypoglycemic potential in patients with type 1 and type 2 diabetes [52]. The dichloromethane fraction of *Kalanchoe pinnata* acts as a potent insulin secretagogue, lowering fasting blood glucose from 228 mg/dL to 116 mg/dL when treated with 10 mg/kg body weight of the DCM fraction [53]. The ethanolic extract of *O. japonicus* significantly lowered hypoglycemic and hypolipidemic effects in STZ-induced diabetic rats [54]. Research on natural α-glucosidase inhibitors from plants aims to develop medicines with fewer unwanted effects than conventional medications do [55]. This enzyme inhibition demonstrated that *C. capitella* has compounds capable of altering carbohydrate digestion, prompting additional research into its effect on blood glucose levels in vivo.

The present study represents significant new contributions to the phytopharmacology of *C. capitella*. For the first time, side-by-side comparisons of its leaf and flower extracts were performed, revealing a consistent advantage of the flower extract in antibacterial, antioxidant, anticancer, and antidiabetic analyses. The authors trace this superior bioactivity directly to the markedly higher levels of phenolics and flavonoids in flowers, providing a clear phytochemical rationale for the observed advantages. They further placed the plant in a regional context by comparing its potency to that of other medicinal species studied in Saudi Arabia and thus incorporated a nonnative species into the local ethnopharmacological landscape.

However, there are significant limitations to the study. To ensure consistency in retention times and spectral matches, each GC-MS run for both CCLE and CCIE should be performed in triplicate. To validate the recently found chemicals, more research is required. It is necessary to utilize linear interpolation concerning the retention lengths of two common n-alkane mixes (C_8_–C_20_ and C_21_–C_40_) to calculate the retention indices of the components. Assays using crude extracts indicate that activity arises from complex mixtures of phytochemicals, making attribution of activity to individual constituents problematic. Chlorophyll removal process is needed to decrease interference in analytical and biological assays. In vitro models, although informative, cannot replicate the pharmacokinetics and metabolic processes of a living organism.

## 5. Conclusions

*C. capitella* exhibits promising multitarget biological activities. CCIE continuously demonstrates strong antibacterial, antioxidant, cytotoxic, and antidiabetic properties because of its high phenolic and flavonoid concentrations. The results further confirm the ethnopharmacological importance of the Crassulaceae family and identify *C. capitella* as a promising candidate for future phytochemical and pharmacological investigations.

## Figures and Tables

**Table 1:** GC/MS compounds in the leaves of CCLE.

Peak	RT	Area	Area%	Name	MF	MW	q-Ions	Classification
1	9.07	39,431,389	1.37	β-Terpinyl acetate	C12H2O2	196	136؛121؛93	Monoterpenoids
2	14.15	7,229,089	0.25	Falcarinol	C17H24O	244	159؛129؛115	Fatty alcohols
3	14.51	13,298,334	0.46	2-Tridecene, (Z)-	C13H26	182	182؛97؛55	Unsaturated aliphatic hydrocarbons
4	21.35	15,480,975	0.54	Phenol, 2,6-bis(1,1-dimethylethyl)-	C14H22O	206	206؛191	Phenylpropanes
5	25.09	7,763,895	0.27	2,4-Di-tert-butylphenol	C14H22O	206	206؛191؛57	Phenylpropanes
6	27.52	17,416,714	0.61	2-Hexadecanol	C16H34O	242	242؛196؛69؛55	Fatty alcohols
7	30.96	18,859,834	0.65	11-Dodecenoic acid, 10-hydroxy-, methyl ester	C13H24O3	228	210؛172؛87؛57	Hydroxy acids and derivatives
8	32.61	9,894,501	0.34	Estra-1,3,5(10)-trien-17β-ol	C18H24O	256	256؛185؛57	Steroids and steroid derivatives
9	33.08	13,401,063	0.46	1-Hexadecanol, 2-methyl-	C17H36O	256	238؛228؛111؛55	Fatty alcohols
10	34.19	50,447,383	1.75	Neophytadiene	C20H38	278	278؛263؛123؛68	Sesquiterpenoids
11	34.82	6,468,493	0.22	Z,E-2,13-Octadecadien-1-ol	C18H34O	266	256؛248؛96؛55	Fatty alcohols
12	35.28	17,176,823	0.59	Desulphosinigrin	C10H17NO6S	279	262؛145؛73؛60	Carbohydrates and carbohydrate conjugates
13	35.84	5,300,915	0.18	7,9-Di-tert-butyl-1-oxaspiro(4,5)deca-6,9-diene-2,8-dione	C17H24O3	276	276؛261؛205	Gamma butyrolactones
14	36.90	50,161,836	1.74	2-Hexadecenoic acid, methyl ester, (E)-	C17H32O2	268	237؛194؛113؛87	Fatty acid esters
15	37.63	52,882,053	1.83	n-Hexadecanoic acid	C16H32O2	256	256؛213؛129؛73	Fatty acids and conjugates
16	38.15	42,950,679	1.49	Hexadecanoic acid, ethyl ester	C18H36O2	284	284؛239؛101؛88	Fatty acid esters
17	40.84	88,459,573	3.07	Phytol	C20H40O	296	296؛123؛71	Diterpenoids
18	41.71	85,640,092	2.98	cis-Linoleic acid	C18H32O2	280	280؛124؛67	Lineolic acids and derivatives
19	42.26	16,551,066	0.57	[1,1′-Bicyclopropyl]-2-octanoic acid, 2′-hexyl-, methyl ester	C21H38O2	322	322؛291؛73	Fatty acid esters
20	42.76	3,243,239	0.11	Oxiraneoctanoic acid, 3-octyl-, cis-	C18H34O3	298	298؛185؛155	Lineolic acids and derivatives
21	43.39	6,100,049	0.21	Linolenic acid, methyl ester	C21H36O4	352	352؛261؛155؛79	Lineolic acids and derivatives
22	44.407	20,170,336	0.701116	2H-Pyran, 2-(7-heptadecynyloxy)tetrahydro-	C22H40O2	336	334؛231؛91؛55	Oxanes
23	46.21	33,445,205	1.162	β-Sitosterol	C29H50O	414	414؛396؛213؛55	Steroids and steroid derivatives
24	46.65	121,315,019	4.21	γ-Sitosterol	C29H50O	414	414؛396؛213؛55؛43	Steroids and steroid derivatives
25	48.87	56,155,458	1.95	Propanoic acid, 2-methyl-, (dodecahydro-6a-hydroxy-9a-methyl-3-methylene-2,9-dioxoazuleno [4,5-b]furan	C19H26O6	350	262؛250؛232	Sesquiterpene lactones
26	49.39	35,380,759	1.22	cis-11-Eicosenoic acid	C20H38O2	310	310؛292؛55	Long-chain fatty acids
27	50.05	53,839,532	1.87	i-Propyl 5,8,11,14,17-eicosapentaenoate	C23H36O2	344	301؛201؛175؛79	Fatty acid methyl esters
28	51.11	11,271,888	0.39	2-[4-methyl-6-(2,6,6-trimethylcyclohex-1-enyl)hexa-1,3,5-trienyl]cyclohex-1-en-1-carboxaldehyde	C23H32O	324	324؛173؛135؛43	Retinoids
29	52.67	85,161,002	2.96	Vitamin E	C29H50O2	430	430؛165	Vitamin E compounds
30	52.81	116,970,643	4.06	Betulin	C30H50O2	442	442؛411	Triterpenoids
31	53.64	878,250,217	30.52	Betulinaldehyde	C30H48O2	440	440؛207؛189	Triterpenoids
32	54.02	568,962,985	19.78	Lupeol	C30H50O	426	426؛218؛189؛95	Triterpenoids
33	55.12	188,984,461	6.56	Oleamide	C18H35NO	281	281؛264؛72؛59	Fatty amides
34	56.04	52,568,959	1.827	Cycloartenol acetate	C33H54O3	498	498؛438؛423	Cycloartanols and derivatives
35	56.41	7,963,550	0.27	Cholest-22-ene-21-ol, 3,5-dehydro-6-methoxy-, pivalate	C33H54O3	498	498؛499؛283؛85؛57	Cholestane steroids
36	58.64	38,477,491	1.33	Stigmasterol	C29H48O	412	412؛351؛300؛255؛55	Steroids and steroid derivatives
37	60.21	39,813,652	1.38	δ-Tocopherol	C27H46O2	402	402؛177؛137	Quinone and hydroquinone lipids

Note: Retention time (RT), molecular formula (MF), and molecular weight (MW).

**Table 2:** GC/MS compounds in CCIE.

Peak	RT	Area	Area%	Name	MF	MW	q-Ions	Classification
1	9.04	36,114,417	4.07	β-Terpinyl acetate	C12H2O2	196	136؛121؛93	Monoterpenoids
2	14.49	8,063,277	0.90	2-Undecanol	C11H24O	172	171؛154؛126؛45	Fatty alcohols
3	21.35	6,918,755	0.77	Phenol, 2,6-bis(1,1-dimethylethyl)-	C14H22O	206	206؛191	Phenylpropanes
4	25.07	4,419,966	0.49	2,4-Di-tert-butylphenol	C14H22O	206	206؛191؛57	Phenylpropanes
5	27.51	14,987,381	1.68	Olealdehyde	C18H34O	266	266؛248؛182؛69؛55	Fatty aldehydes
6	31.02	2,637,007	0.29	7-Ethyl-4-decen-6-one	C12H22O	182	126؛97؛55	Carbonyl compounds
7	32.59	7,830,307	0.88	Estra-1,3,5(10)-trien-17β-ol	C18H24O	256	256؛185؛57	Steroids and steroid derivatives
8	33.07	11,437,702	1.28	1-Hexadecanol, 2-methyl-	C17H36O	256	238؛228؛111؛55	Fatty alcohols
9	34.18	11,473,302	1.29	10-Methyl-E-11-tridecen-1-ol propionate	C17H32O2	268	268؛253؛96؛82؛57	Fatty alcohol esters
10	34.52	2,098,725	0.23	7-Methyl-Z-tetradecen-1-ol acetate	C17H32O2	268	268؛129؛55؛43	Fatty alcohol esters
11	34.81	1,064,966	0.12	Heptadecyl acetate	C19H38O2	298	238؛210؛87؛43	Fatty alcohol esters
12	35.27	3,185,425	0.35	12-Methyl-E,E-2,13-octadecadien-1-ol	C19H36O	280	280؛262؛220؛211؛55	Fatty alcohols
13	35.83	8,369,893	0.94	7,9-Di-tert-butyl-1-oxaspiro(4,5)deca-6,9-diene-2,8-dione	C17H24O3	276	276؛261؛217؛205؛175؛57	Gamma butyrolactones
14	36.46	1,636,582	0.18	11-Dodecenoic acid, 10-hydroxy-, methyl ester	C13H24O3	228	172؛143؛87؛57	Hydroxy acids and derivatives
15	36.91	2,509,817	0.28	Theaspirane (Isomer 2)	C13H24O2	212	212؛170؛126؛85	Tetrahydrofurans
16	37.12	1,606,989	0.18	Hexadecenoic acid, Z-11-	C16H30O2	254	254؛236؛97؛83؛69؛55	Long-chain fatty acids
17	37.61	69,490,912	7.84	n-Hexadecanoic acid	C16H32O2	256	256؛213؛129؛73	Fatty acids and conjugates
18	38.13	8,992,293	1.01	Hexadecanoic acid, ethyl ester	C18H36O2	284	284؛239؛101؛88	Fatty acid esters
19	40.27	2,563,673	0.28	Palmitoleic acid	C16H30O2	254	254؛236؛152؛137؛55	Long-chain fatty acids
20	40.41	2,959,956	0.33	9,12-Octadecadienoyl chloride, (Z,Z)-	C18H31ClO	298	298؛262؛81؛67	Acyl chlorides
21	40.58	1,813,772	0.20	Phytol	C20H40O	296	296؛123؛71	Diterpenoids
22	40.81	11,585,198	1.31	Erucic acid	C22H42O2	338	320؛181	Long-chain fatty acids
23	41.61	128,284,519	14.45	cis-Linoleic acid	C18H32O2	280	280؛124؛67	Lineolic acids and derivatives
24	41.72	144,352,463	16.26	cis-9-Tetradecenoic acid, isobutyl ester	C18H34O2	282	282؛209؛57	Very long-chain fatty acids
25	41.93	9,212,060	1.03	17-Octadecynoic acid	C18H32O2	280	123؛109؛81؛55	Fatty acid esters
26	42.11	3,359,860	0.37	Ethanol, 2-(9,12-octadecadienyloxy)-, (Z,Z)-	C20H38O2	310	310؛248؛67	Long-chain fatty acids
27	42.23	20,556,698	2.31	Oleic Acid	C18H34O2	282	282؛264؛220؛55؛41	Dialkyl ethers
28	42.74	4,769,101	0.53	Oxiraneoctanoic acid, 3-octyl-, cis-	C18H34O3	298	298؛185؛155	Lineolic acids and derivatives
29	43.39	37,279,887	4.21	Linolenic acid, methyl ester	C21H36O4	352	352؛261؛155؛79	Lineolic acids and derivatives
30	44.78	3,007,109	0.33	5,8,11,14-Eicosatetraenoic acid, methyl ester, (all-Z)-	C21H34O2	318	318؛203؛175؛150؛91؛79	Fatty acid methyl esters
31	44.89	1,819,417	0.21	cis-5,8,11-Eicosatrienoic acid, methyl ester	C21H36O2	320	320؛161؛106؛93؛79	Fatty acid methyl esters
32	47.14	4,147,120	0.46	6,9,12,15-Docosatetraenoic acid, methyl ester	C23H38O2	346	346؛264؛235؛149	Fatty acid methyl esters
33	47.63	6,755,888	0.76	psi.-Diosgenin	C27H42O3	414	414؛396؛357؛317	Steroids and steroid derivatives
34	48.38	5,911,898	0.66	9,12,15-Octadecatrienoic acid, 2-(acetyloxy)-1-[(acetyloxy)methyl]ethyl	C25H40O6	436	171؛159؛43	Lineolic acids and derivatives
35	48.51	1,462,102	0.16	Tricyclo [20.8.0.0(7,16)]triacontane, 1(22),7(16)-diepoxy-	C30H52O2	440	444؛426؛95؛55	Epoxides
36	48.94	3,884,597	0.437	Propanoic acid, 2-methyl-, (dodecahydro-6a-hydroxy-9a-methyl-3-methylene-2,9-dioxoazuleno [4,5-b]furan	C19H26O6	350	262؛250؛232	Sesquiterpene lactones
37	49.12	3,668,293	0.41	cis-11-Eicosenoic acid	C20H38O2	310	310؛292؛55	Long-chain fatty acids
38	49.41	6,580,710	0.74	i-Propyl 5,8,11,14,17-eicosapentaenoate	C23H36O2	344	301؛201؛175؛79	Fatty acid esters
39	49.88	20,523,091	2.31	Cholestan-3-ol, 2-methylene-, (3β,5α)-	C28H48O	400	400؛329؛315؛95؛69	Steroids and steroid derivatives
40	51.12	10,221,715	1.15	Cholest-22-ene-21-ol, 3,5-dehydro-6-methoxy-, pivalate	C33H54O3	498	498؛283؛85؛57	Cholestane steroids
41	52.31	9,361,266	1.05	Androstan-17-one, 3-ethyl-3-hydroxy-, (5α)-	C21H34O2	318	300؛282؛271؛105؛91	Steroids and steroid derivatives
42	52.47	24,870,581	2.80	Spiro[androstane-3,2′-thiazolidine], (5α)-	C21H35NS	333	332؛286؛273؛258	Steroids and steroid derivatives
43	53.02	4,601,229	0.51	Eicosanoic acid, phenylmethyl ester	C27H46O2	402	311؛281؛108؛91	Benzene and substituted derivatives
44	53.13	9,611,225	1.08	Hexadecane, 1,1-bis(dodecyloxy	C40H82O2	594	440؛408؛257؛57؛43	Ethers
45	53.62	3,299,552	0.37	2-Monolinolenin, 2TMS derivative	C25H40O6	436	159؛43	Lineolic acids and derivatives
46	53.84	2,362,268	0.26	Lupeol	C30H50O	426	426؛218؛189؛95	Triterpenoids
47	55.10	70,850,428	7.98	Oleamide	C18H35NO	281	281؛72؛59	Fatty amides
48	56.52	21,896,133	2.46	Campesterol	C28H48O	400	400؛382؛315	Steroids and steroid derivatives
49	58.46	60,150,775	6.77	Stigmasterol	C29H48O	412	412؛351؛300؛255؛55	Stigmastanes and derivatives
50	59.05	33,997,766	3.83	Campesterol	C28H48O	400	400؛382؛367	Steroids and steroid derivatives
51	60.15	8,701,951	0.98	δ-Tocopherol	C27H46O2	402	402؛177؛137	Quinone and hydroquinone lipids

Note: Retention time (RT), molecular formula (MF), and molecular weight (MW).

**Table 3:** Inhibitory zone (mm), MIC (μg/mL), and MBC (μg/mL) of CCLE.

Bacterium/Dilution	Positive Control	800 μg/mL	400 μg/mL	200 μg/mL	100 μg/mL	MIC (μg/mL)	MBC (μg/mL)
*S. aureus*	24 ± 0.00	23 ± 0.00 *	20 ± 0.00 *	17 ± 0.00 *	12 ± 0.00 *	6.25 ± 0.00	12.50 ± 0.00
*S. epidermidis*	30 ± 0.00	24 ± 0.00 *	20 ± 0.00 *	16 ± 0.00 *	11 ± 0.00 *	12.50 ± 0.00	25 ± 0.00
*B. subtilis*	21 ± 0.00	21 ± 0.00 *	19 ± 0.00 *	15 ± 0.00 *	12 ± 0.00 *	25 ± 0.00	50 ± 0.00
*E. coli*	32 ± 0.00	24 ± 0.00 *	21 ± 0.00 *	17 ± 0.00 *	12 ± 0.00 *	25 ± 0.00	50 ± 0.00
*K. pneumoniae*	23 ± 0.00	20 ± 0.00 *	15 ± 0.00 *	11 ± 0.00 *	9 ± 10.00	50 ± 0.00	100 ± 0.00
*P. aeruginosa*	21 ± 0.00	22 ± 0.00 *	20 ± 0.00 *	15 ± 0.00 *	12 ± 0.00 *	25 ± 0.00	50 ± 0.00

Note The minimum inhibitory concentration (MIC) and minimum bactericidal concentration (MBC). The reported values are shown in triplicate as the means ± SDs. The results revealed a statistically significant decrease from the positive control (25 µg/mL chloramphenicol), as indicated by * = *p* < 0.05.

**Table 4:** Inhibitory zone (mm), MIC (μg/mL), and MBC (μg/mL) of CCIE.

Bacterium/Dilution	Positive Control	800 μg/mL	400 μg/mL	200 μg/mL	100 μg/mL	MIC (μg/mL)	MBC (μg/mL)
*S. aureus*	24 ± 0.00	23 ± 0.00 *	19 ± 0.00 *	16 ± 0.00 *	14 ± 0.00 *	6.25 ± 0.00	12.50 ± 0.00
*S. epidermidis*	30 ± 0.00	25 ± 0.00 *	20 ± 0.00 *	18 ± 0.00 *	15 ± 0.00 *	12.50 ± 0.00	50.00 ± 0.00
*B. subtilis*	21 ± 0.00	20 ± 0.00 *	17 ± 0.00 *	15 ± 0.00 *	13 ± 0.00 *	12.50 ± 0.00	50 ± 0.00
*E. coli*	32 ± 0.00	22 ± 0.00 *	19 ± 0.00 *	15 ± 0.00 *	10 ± 0.00 *	12.50 ± 0.00	50.00 ± 0.00
*K. pneumoniae*	23 ± 0.00	19 ± 0.00 *	17 ± 0.00 *	15 ± 0.00 *	11 ± 10.00 *	25 ± 0.00	50 ± 0.00
*P. aeruginosa*	21 ± 0.00	26 ± 0.00	22 ± 0.00 *	18 ± 0.00 *	15 ± 0.00 *	25 ± 0.00	50 ± 0.00

Note The minimum inhibitory concentration (MIC) and minimum bactericidal concentration (MBC). The reported values are shown in triplicate as the means ± SDs. The results revealed a statistically significant decrease from the positive control (25 µg/mL chloramphenicol), as indicated by * = *p* < 0.05.

## Data Availability

The original contributions presented in this study are included in the article. Further inquiries can be directed to the corresponding authors.
